# Dynamic Characteristics of Coupled Dual-Oscillator Piezoelectric Vibration Energy Harvester with External Magnet

**DOI:** 10.3390/mi17030356

**Published:** 2026-03-14

**Authors:** Zejing Huang, Huabiao Zhang, Yang Yang, Lijuan Zhang, Xinye Li, Yu Sheng

**Affiliations:** 1 School of Mechanical Engineering, Tianjin University of Commerce, Tianjin 300134, China; 2 School of Automobile and Transportation, Tianjin University of Technology and Education, Tianjin 300134, China; 3 School of Mechanical Engineering, Hebei University of Technology, Tianjin 300134, China; 4 National Key Labortory of Particle Transport and Separation Technology, Tianjin 300171, China

**Keywords:** dual-oscillator piezoelectric energy harvester, external magnet, dynamic characteristics, harvesting performance, magnet spacing

## Abstract

Magnetic nonlinearity and multi-oscillator coupling are commonly employed to improve the performance of energy harvesters. This study integrates both mechanisms to propose a nested dual-oscillator coupled piezoelectric energy harvester with an external magnet, investigating both repulsive and attractive interactions between the two oscillators. The influence of parameters on static/dynamic characteristics and harvesting performance is analyzed. For the repulsive-type harvester, the response under weak excitation is characterized by small-amplitude in-phase motion within potential wells; under strong excitation, one oscillator exhibits a large-amplitude response while the other remains nearly quiescent, and non-periodic responses may occur. Large magnet spacings effectively enhance the bandwidth and output power. The attractive-type harvester primarily shows in-phase periodic motion, though non-periodic behavior may appear under strong excitation. Small moving-magnet spacing combined with large external-magnet spacing can significantly boost bandwidth and power output. In both configurations, performance declines as the external-magnet spacing exceeds an optimal range. The repulsive-type harvester features a wider potential well, performing well under weak excitation, whereas the attractive-type, with vibration modes aligned to the potential well profile, is more likely to generate large-amplitude responses under strong excitation. Experimental results show excellent agreement with simulation data, confirming the reliability of the proposed design.

## 1. Introduction

Piezoelectric energy harvesters (PEHs) efficiently convert ambient mechanical vibrations into electrical energy via the direct piezoelectric effect. Owing to their simple architecture, low fabrication cost, high reliability, and substantial power density, PEHs exhibit significant potential for powering self-sustained systems such as wireless sensor networks, smart instrumentation, and wearable biomedical devices [1]. Consequently, enhancing the performance of PEHs has become a focal point in recent research.

To overcome the narrow bandwidth limitation of conventional linear PEHs—particularly in low-frequency environments—researchers have introduced magnetic coupling mechanisms to establish multistable dynamic systems, thereby achieving broadband energy harvesting. These configurations leverage nonlinear potential landscapes to promote inter-well transitions, significantly expanding the effective operating frequency range and improving energy conversion efficiency.

Samuel et al. [2] investigated a bistable inertial oscillator composed of a piezoelectric cantilever beam with an end-mounted permanent magnet interacting with fixed external magnets. The system was rigorously characterized through analytical modeling, numerical simulation, and experimental validation, demonstrating enhanced energy harvesting performance under stochastic and harmonic excitations. Leng et al. [3] explored a tristable PEH configuration in which a piezoelectric cantilever with a tip magnet was influenced by two fixed external magnets. This arrangement generates three stable equilibrium positions, enabling large-amplitude inter-well oscillations and broadband voltage responses under low-frequency excitation. Zhou et al. [4,5] proposed a quadstable energy harvester incorporating a piezoelectric cantilever beam with a tip magnet and three fixed external magnets. By tuning the spacing between the magnets, four stable equilibrium states can be established, further broadening the dynamic response bandwidth and promoting multi-modal energy capture. Ju et al. [6] systematically analyzed the bifurcation mechanisms of asymmetric static equilibria in tri- and quadstable PEHs, revealing that geometric nonlinearity introduces higher-order (third- and fifth-order) stiffness terms, while the gravitational effect of the magnetic mass leads to asymmetry in the potential wells—both of which critically influence the system’s dynamic behavior. Wang et al. [7] examined the influence of asymmetric potential barriers on the dynamic response and effective bandwidth, highlighting the role of asymmetry in facilitating low-threshold inter-well transitions and enhancing energy harvesting under suboptimal excitation conditions. Mei et al. [8,9] extended the application of tri- and quadstable PEHs to low-frequency rotational motion, investigating how gravity and magnetic positioning affect potential barrier modulation. They employed perturbation methods to analytically describe the oscillatory behavior of the piezoelectric beam near both zero and nonzero equilibrium points, providing theoretical insight into rotational energy harvesting dynamics. Zheng et al. [10] developed a PEH with a time-varying and asymmetric potential well, realized by mounting a pair of external magnets on compliant springs. This design enables adaptive potential shaping under dynamic loading, enhancing responsiveness across a wider frequency spectrum. Man et al. [11] studied the performance enhancement of a tristable cantilever PEH using vertical and rotational elastic amplifiers, demonstrating that mechanical amplification mechanisms can further boost output power by increasing effective strain in the piezoelectric layer. Zhang et al. [12,13] designed a magnetically coupled tristable PEH based on a hybrid structure combining straight and curved beams. This configuration supports multi-directional excitation and wide-frequency energy harvesting, making it particularly suitable for real-world environments with complex vibration profiles.

The coupling of two or more oscillators can significantly alter the system’s potential energy landscape, thereby broadening the effective operating bandwidth and enhancing the output power. Kim et al. [14] introduced phase-dependent dynamic potential in a two-degree-of-freedom (2-DOF) magnetically coupled bistable PEH, identifying two distinct dynamic regimes: an out-of-phase mode—exhibiting double-well dynamics in frequency-response and power-output characteristics—and an in-phase mode, which displays single-well behavior despite the system’s static bistability. These regimes emerge within the first and second primary intra-well resonance frequency regions. Wu et al. [15] proposed a nonlinear design comprising two asymmetric cantilever beams with a 1:3 resonant frequency ratio, incorporating two vibrational degrees of freedom (DOFs). The primary DOF captures ambient low-frequency vibrations, while the secondary DOF undergoes strong excitation at its higher resonant frequency (three times the input frequency), enabling efficient frequency up-conversion. Zou et al. [16] developed a magnetically coupled 2-DOF vibration PEH tailored for rotary motion, consisting of two inverted piezoelectric cantilever beams oriented toward a rotating shaft. Under rotation, centrifugal forces induce large-amplitude oscillations even at low rotational speeds, demonstrating high suitability for low-speed rotational energy harvesting and multi-frequency band operation. Noh et al. [17] optimized the external load resistance in a magnetically coupled 2-DOF bistable PEH to maximize power output, accounting for third-harmonic distortion in the forced response. They further investigated the system’s nonlinear dynamics using harmonic balance analysis (HBA), identifying all periodic solution branches to elucidate complex dynamic behaviors [18]. Accurate truncation of the HBA solution is essential—unlike conventional single-DOF magnetically coupled bistable PEHs where the fundamental harmonic adequately captures the steady-state response, higher-order harmonics play a critical role in 2-DOF systems. In addition to magnetic coupling, elastic coupling represents another fundamental mechanism for oscillator interaction. Shim et al. [19] designed a PEH incorporating two elastic supports and four piezoelectric beams with distinct natural frequencies. Elastic coupling through the supports enhances voltage generation across all resonant frequencies and introduces beneficial nonlinearity, thereby broadening the operational bandwidth. Shen et al. [20,21,22,23] proposed a hybrid system consisting of a linear oscillator and a bistable oscillator, elastically coupled via a linear spring. An electromechanical model was established, with the nonlinear restoring force experimentally characterized. Numerical simulations, harmonic analysis, and random-vibration analysis were conducted to evaluate system performance for varying coupling stiffness values.

Energy harvesting based on novel principles is a key research hotspot. Inspired by lung tracheae, Wang et al. [24] proposed a bio-inspired tracheal structure, whose novelty lies in adapting to various excitations by adjusting the steady-state model via rotating gears. Tests show that its tristable to hexastable states yield excellent low-frequency, low-amplitude energy harvesting performance. Zhao et al. [25] presented a self-aligning mechanism similar to a self-spooling pulley for biomechanical energy harvesting. It adaptively aligns internal driving direction with external excitation, improving efficiency and reducing wear. Bolat et al. [26] studied negative Poisson’s ratio (NPR) beams for vibration energy harvesting. The NPR effect reduces stiffness and resonant frequency, and experiments show that NPR beams increase energy output by about 20% over conventional beams.

This paper simultaneously considers magnetic nonlinearity and coupling between dual-oscillators and proposes a nested dual-oscillator coupled PEH with an external magnet. We conduct simulations considering both repulsive and attractive interactions between the two oscillators and analyze the influence of the parameters on the static and dynamic characteristics, as well as on the performance of the PEH. The main structure of this paper is as follows: Section 2 introduces the structure of the dual-oscillator PEH and establishes the nonlinear dynamic equations based on the Lagrange equation and the magnetic dipole model. Section 3 analyzes the effect of magnet spacing on the PEH’s static characteristics. Section 4 investigates the effects of acceleration amplitude, magnet spacing, and excitation frequency on the dynamic characteristics and harvesting performance. Section 5 establishes an experimental prototype of the PEH and experimentally verifies the previous simulation results. Finally, the research of this paper is summarized.

## 2. Structure and Dynamic Model of the PEH

### 2.1. Structure of the PEH

**Figure 1:**
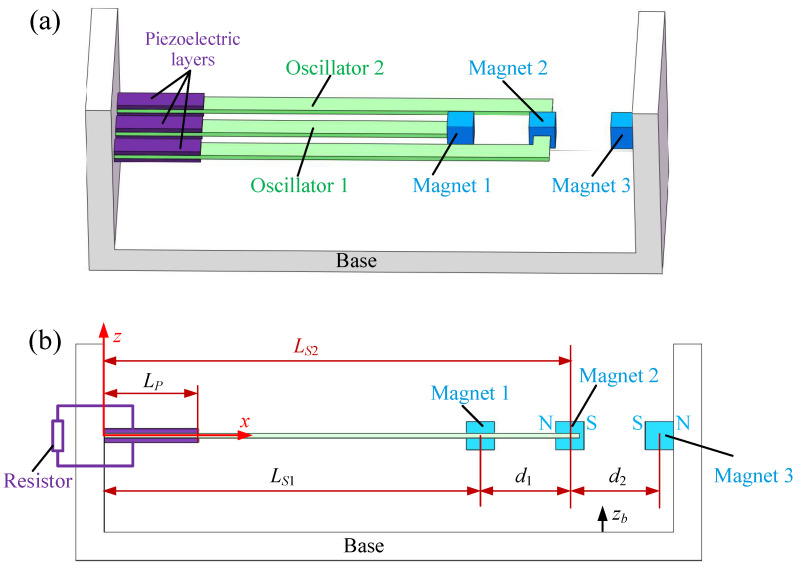
The structure of the dual-oscillator coupled PEH with an external magnet: (**a**) The 3D view. (**b**) The side view.

The dual-oscillator coupled PEH studied in this paper adopts an inner–outer dual cantilever beam structure, with piezoelectric layers symmetrically bonded to the root surfaces of the cantilever beam substrate (see Figure 1). The thickness direction of the piezoelectric layers serves as the polarization direction, with opposite polarizations on the upper and lower layers connected in series with load resistors. Magnets 1 and 2 are installed at the free ends of the two oscillators, while magnet 3 is an external fixed magnet. A repulsive interaction exists between magnets 2 and 3. Regarding the polarity of magnet 1, we consider two cases in our study: the repulsive type, where the right side of magnet 1 is the N-pole, resulting in repulsion between magnets 1 and 2, and the attractive type, where the right side of magnet 1 is the S-pole, leading to attraction between magnets 1 and 2. When the base is subjected to vertical excitation, the oscillators vibrate and the piezoelectric layers convert their strain into electrical energy. Compared to conventional dual-oscillator coupled configurations, the incorporation of an external fixed magnet introduces additional tunable parameters, thereby enhancing the system’s design flexibility and enabling more precise adaptation to complex and dynamically varying excitation conditions, which ultimately leads to improved energy harvesting performance.

### 2.2. Kinetic Energy and Elastic Potential Energy of the PEH

As shown in Figure 1b, we establish a coordinate system with the origin at the center of the oscillators’ roots, where the x-axis points rightward, the z-axis points upward, and the y-axis points outward perpendicular to the plane of the paper. We make the following assumptions: (1) ignoring the geometric nonlinearity of the oscillators; (2) assuming the magnets at the oscillators’ ends are a point mass and ignoring their size and moment of inertia; (3) assuming perfect bonding between the piezoelectric layers and the substrates and ignoring the effects of the adhesive; (4) assuming the deformation of the oscillators satisfies the Euler–Bernoulli conditions; and (5) assuming the electric field intensity of the piezoelectric film is uniformly distributed. Thus, the kinetic energy of the piezoelectric oscillator *k* is

(1) $$\begin{array}{c} T_{k}=\frac{1}{2}\rho_{S}A_{Sk}\int_{0}^{L_{Sk}}{\left[{\dot{w}}_{k}\left(x,t\right)+{\dot{z}}_{b}\right]}^{2}dx+\rho_{P}A_{Pk}\int_{0}^{L_{Pk}}{\left[{\dot{w}}_{k}\left(x,t\right)+{\dot{z}}_{b}\right]}^{2}dx\\ +\frac{1}{2}m_{k}{\left[{\dot{w}}_{k}\left(L_{k},t\right)+{\dot{z}}_{b}\right]}^{2}, \end{array}$$

where the subscript k=1,2 denotes oscillator 1 (the inner beam) and 2 (the outer beam), respectively; this notation is maintained throughout this paper. “·” denotes the derivative with respect to time. $w_{k}\left(x,t\right)$ is the vertical displacement of the oscillators, $z_{b}$ is the base displacement, and $\rho_{S}$ and $\rho_{P}$ are the densities of the substrate and the piezoelectric layer, respectively. $A_{Sk}$ and $A_{Pk}$ are the cross-sectional areas of the substrate and piezoelectric layer of oscillator *k*, respectively. *m* is the mass of the end magnet. Then, the total kinetic energy of the system is

(2) $$T=T_{1}+T_{2}.$$

According to the Euler–Bernoulli beam theory, the axial strain of oscillator *k* is

(3) $$S=-zw_{k}^{\prime \prime }\left(x,t\right),$$

where ″ represents the second derivative with respect to *x*. Thus, the elastic potential energy of the substrate of oscillator *k* can be written as

(4) $$U_{Sk}=\frac{1}{2}C_{S}I_{Sk}\int_{0}^{L_{Sk}}w_{k}^{\prime \prime }{\left(x,t\right)}^{2}dx,$$

where $C_{S}$ is the elastic modulus of the substrate, $I_{Sk}$ is the cross-section moment of inertia of the substrate about the *y*-axis, and

(5) $$I_{Sk}=\frac{1}{12}h_{S}^{3}b_{Sk}.$$

Since the piezoelectric piece satisfies the mechanical clamping and electrical short circuit conditions along the length of the beam, the voltage constitutive equation is

(6) $$\left\{\begin{array}{c} T_{1}=C_{11}S_{1}-e_{31}E_{3},\\ D_{3}=e_{31}S_{1}+\varepsilon_{33}E_{3}, \end{array}\right.$$

where $T_{1}$ and $S_{1}$ represent the mechanical stress and strain of the piezoelectric layer, $E_{3}$ and $D_{3}$ represent the electric field strength and electric displacement, $e_{31}$ is the electromechanical coupling coefficient, $C_{11}$ represents the elastic modulus measured under zero electric field, and $\varepsilon_{33}$ is the dielectric constant of the piezoelectric material under zero strain. Then the electric field strength of the piezoelectric layer is

(7) $$E_{3k}=-\frac{V_{k}}{2h_{P}},$$

where $V_{k}$ is the output voltage and $h_{P}$ is the thickness of the piezoelectric layers. Therefore, the potential energy of the piezoelectric layer of oscillator *k* is

(8) $$\begin{array}{c} U_{Pk}=\frac{1}{2}\sum_{i=1,2}\int_{V_{Pki}}\left(T_{1ki}S_{1ki}-E_{3ki}D_{3ki}\right)dV_{Pki}\\ =\frac{1}{2}C_{11}I_{Pk}\int_{0}^{L_{P}}{\left(w_{k}^{\prime \prime }\right)}^{2}dx-\frac{1}{2}e_{31}V_{k}\left(t\right)b_{Pk}\left(h_{P}+h_{s}\right)\int_{0}^{L_{P}}w_{k}^{\prime \prime }dx-\frac{1}{4}C_{Pk}{V_{k}}^{2}, \end{array}$$

where i=1,2 correspond to the piezoelectric layers above and below the substrate, $L_{P}$ is the length of the piezoelectric layers, $I_{Pk}$ denotes the cross-section moment of inertia of the piezoelectric layer, and $C_{Pk}$ is the capacitance of the piezoelectric layer, where

(9) $$\begin{array}{c} {I_{P}}_{k}=\frac{1}{6}{b_{P}}_{k}h_{P}\left(3{h_{S}}^{2}+6h_{S}h_{P}+4{h_{P}}^{2}\right),\\ C_{Pk}=\frac{L_{P}b_{Pk}\varepsilon_{33}}{h_{P}}. \end{array}$$

The gravitational potential energy of oscillator *k* can be expressed as

(10) $$U_{gk}=\rho_{S}A_{Sk}g\int_{0}^{L_{Sk}}w_{k}\left(x,t\right)dx+2\rho_{P}A_{Pk}g\int_{0}^{L_{P}}w_{k}\left(x,t\right)dx+mgw_{k}\left(L_{Sk},t\right).$$

### 2.3. Magnetic Potential Energy of the PEH

**Figure 2:**
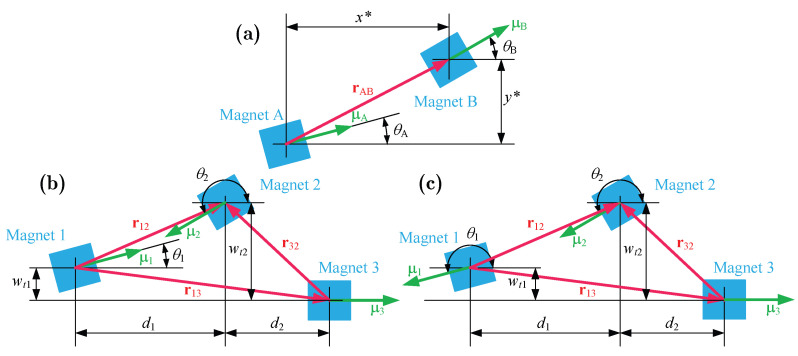
The magnetic dipole model: (**a**) The two-magnet model. (**b**) The repulsive-type PEH model. (**c**) The attractive-type PEH model.

To facilitate the calculation, this paper assumes that the magnetic field is uniformly distributed and treats the magnets as tiny magnetic dots. The magnetic dipole model is used to analyze magnet–magnet interactions. The magnetic potential energy between the two magnets shown in Figure 2a is calculated as follows [2]:

For two magnetic dipoles A and B, the direction vector from A to B is

(11) $$r_{AB}=\left[\begin{array}{c} x^{*}\\ y^{*} \end{array}\right],$$

The magnetic moment vectors of A and B are

(12) $$\mu_{A}=M_{A}V_{A}\left[\begin{array}{c} \cos\theta_{A}\\ \sin\theta_{A} \end{array}\right],\mu_{B}=M_{B}V_{B}\left[\begin{array}{c} \cos\theta_{B}\\ \sin\theta_{B} \end{array}\right],$$

where $M_{A}=M_{B}=M$ is the magnetization of the magnet and $V_{A}=V_{B}=V$ represents the volume of the magnet. $\theta_{A}$ and $\theta_{B}$ are the angles between the vectors and the horizontal, respectively. Magnetic flux density generated by magnetic dipole A at B is

(13) $$\begin{array}{c} B_{AB}=-\frac{\mu_{0}}{4\pi }\nabla \frac{\mu_{A}\cdot r_{AB}}{{{∥r_{AB}∥}_{2}}^{3}}\\ =\frac{\mu_{0}MV}{4\pi {\left({x^{*}}^{2}+{y^{*}}^{2}\right)}^{5/2}}\left[\begin{array}{c} \left(2{x^{*}}^{2}-{y^{*}}^{2}\right)\cos\theta_{A}+3x^{*}y^{*}\sin\theta_{A}\\ 3x^{*}y^{*}\cos\theta_{A}+\left(2{y^{*}}^{2}-{x^{*}}^{2}\right)\sin\theta_{A} \end{array}\right], \end{array}$$

where $\mu_{0}=4\pi \times 10^{-7}\;H/m$ is vacuum permeability, ${\left\|\right\|}_{2}$ is the two-norm operation, and ∇ is the vector gradient operator. The magnetic potential energy $U_{mAB}$ between A and B is

(14) $$\begin{array}{c} U_{mAB}=-B_{AB}\cdot \mu_{B}\\ =-\frac{\mu_{0}M^{2}V^{2}}{4\pi {\left({x^{*}}^{2}+{y^{*}}^{2}\right)}^{5/2}}[\left({y^{*}}^{2}-2{x^{*}}^{2}\right)\cos\theta_{A}\cos\theta_{B}-3x^{*}y^{*}\sin\theta_{A}\cos\theta_{B}\\ +3x^{*}y^{*}\cos\theta_{A}\sin\theta_{B}+\left(2{y^{*}}^{2}-{x^{*}}^{2}\right)\sin\theta_{A}\sin\theta_{B}], \end{array}$$

For the PEH studied in this paper (see Figure 2b,c), only the direction vector and magnetic moment vector between the magnets are changed. For the three magnets, there are

(15) $$r_{12}=\left[\begin{array}{c} d_{1}\\ w_{t2}-w_{t1} \end{array}\right],r_{13}=\left[\begin{array}{c} d_{1}+d_{2}\\ -w_{t1} \end{array}\right],r_{23}=\left[\begin{array}{c} -d_{2}\\ w_{t2} \end{array}\right],\;\mu_{1}=M_{1}V_{1}\left[\begin{array}{c} \cos\theta_{1}\\ \sin\theta_{1} \end{array}\right],\mu_{2}=M_{2}V_{2}\left[\begin{array}{c} \cos\theta_{2}\\ \sin\theta_{2} \end{array}\right],\mu_{3}=M_{3}V_{3}\left[\begin{array}{c} 1\\ 0 \end{array}\right],$$

where $w_{tk}$ is the displacement of the free end of oscillator *k*. $\theta_{1}$ and $\theta_{2}$ can be determined by the rotation angles of the free ends of the oscillators.

(16) $$\begin{array}{c} \cos\theta_{1}=\pm \frac{1}{\sqrt{1+{{w^{\prime }}_{t1}}^{2}}},\sin\theta_{1}=\pm \frac{{w^{\prime }}_{t1}}{\sqrt{1+{{w^{\prime }}_{t1}}^{2}}},\\ \cos\theta_{2}=\frac{1}{\sqrt{1+{{w^{\prime }}_{t2}}^{2}}},\sin\theta_{2}=\frac{{w^{\prime }}_{t2}}{\sqrt{1+{{w^{\prime }}_{t2}}^{2}}}. \end{array}$$

The sign ± in Equation (16) is taken as positive for the repulsive-type PEH and negative for the attractive-type PEH. Hence, the total magnetic potential energy can be expressed as

(17) $$U_{m}=U_{m12}+U_{m13}+U_{m23}.$$

The total potential energy of the system is composed of three distinct contributions: the elastic potential energy, the gravitational potential energy, and the magnetic potential energy, as detailed below.

(18) $$U=U_{k1}+U_{k2}+U_{g1}+U_{g2}+U_{m}.$$

### 2.4. Modal Mode of the Piezoelectric Oscillators

The environmental vibration frequency of the PEH is usually very low, so this paper only considers the first-order mode of the piezoelectric oscillator, and the displacement function of the piezoelectric oscillator can be expressed as

(19) $$w_{k}\left(x,t\right)=\varphi_{k}\left(x\right)r_{k}\left(t\right),$$

where $\varphi_{k}\left(x\right)$ and $r_{k}\left(t\right)$ are the first-order mode shape function and modal coordinates of oscillator *k*, respectively. Since the cross-section shape of the piezoelectric oscillator suddenly changes at the position $x=L_{P}$, we set the mode shape function to a piecewise form [27].

(20) $$\begin{array}{c} \varphi_{1}=\left\{\begin{array}{cc} \varphi_{11}=c_{1}\cos\beta_{1}x+c_{2}\cos\beta_{1}x+c_{3}\cosh\beta_{1}x+c_{4}\sinh\beta_{1}x & 0\leq x\leq L_{P}\\ \varphi_{12}=d_{1}\cos\beta_{2}x+d_{2}\cos\beta_{2}x+d_{3}\cosh\beta_{2}x+d_{4}\sinh\beta_{2}x & L_{P}<x\leq L_{S1}, \end{array}\right.\\ \varphi_{2}=\left\{\begin{array}{cc} \varphi_{21}=c_{5}\cos\beta_{3}x+c_{6}\cos\beta_{3}x+c_{7}\cosh\beta_{3}x+c_{8}\sinh\beta_{3}x & 0\leq x\leq L_{P}\\ \varphi_{22}=d_{5}\cos\beta_{4}x+d_{6}\cos\beta_{4}x+d_{7}\cosh\beta_{4}x+d_{8}\sinh\beta_{4}x & L_{P}<x\leq L_{S2}, \end{array}\right. \end{array}$$

where

(21) $$\begin{array}{c} \beta_{1}={\omega_{1}}^{2}\left(\rho_{S}A_{S1}+\rho_{P}A_{P1}\right)/\left(C_{S}I_{S1}+C_{11}I_{P1}\right),\\ \beta_{2}={\omega_{1}}^{2}\rho_{S}A_{S1}/C_{S}I_{S1},\\ \beta_{3}={\omega_{2}}^{2}\left(\rho_{S}A_{S2}+\rho_{P}A_{P2}\right)/\left(C_{S}I_{S2}+C_{11}I_{P2}\right),\\ \beta_{4}={\omega_{2}}^{2}\rho_{S}A_{S2}/C_{S}I_{S2}, \end{array}$$

Consider the following boundary conditions: (1) the deflection and rotation angles at the fixed end are zero; (2) the bending moment at the free end is zero and the shear force is equal to the inertia force of the concentrated mass; and (3) at the abrupt connection position, the displacement, rotation angle, bending moment, and shear force of the beam are continuous, so that

(22) $$\begin{array}{c} \varphi_{k1}\left(0\right)=0,\\ {\varphi^{\prime }}_{k1}\left(0\right)=0,\\ \left(C_{S}I_{Sk}+C_{11}I_{Pk}\right)\varphi_{k1}^{\prime \prime }\left(L_{P}\right)=C_{S}I_{Sk}\varphi_{k2}^{\prime \prime }\left(L_{P}\right),\\ \left(C_{S}I_{Sk}+C_{11}I_{Pk}\right)\varphi_{k1}^{\prime \prime \prime }\left(L_{P}\right)=C_{S}I_{Sk}\varphi_{k2}^{\prime \prime \prime }\left(L_{P}\right),\\ \varphi_{k2}^{\prime \prime }\left(L_{S}\right)=0,\\ C_{S}I_{S}\varphi_{k2}^{\prime \prime \prime }\left(L_{S}\right)=-m{\omega_{k}}^{2}\varphi_{k2}\left(L_{S}\right), \end{array}$$

Solving the equations can determine the natural frequency $\omega_{k}\left(k=1,2\right)$ and the undetermined coefficients $c_{i},d_{i},(i$ = 1–8), and can obtain the first-order mode shape function of the oscillators.

### 2.5. Dynamic Equations of the PEH

The dynamic equations of the PEH can be obtained from the following equations based on the Lagrange equation:

(23) $$\begin{array}{c} \frac{d}{dt}\frac{\partial L}{\partial {\dot{r}}_{k}}-\frac{\partial L}{\partial r_{k}}=F_{k},\\ \frac{d}{dt}\frac{\partial L_{k}}{\partial {\dot{\lambda }}_{k}}-\frac{\partial L_{k}}{\partial \lambda_{k}}=Q_{k}, \end{array}$$

where $\lambda_{k}$ is the flux linkage, ${\dot{\lambda }}_{k}=V_{k}$, L=T−U is the Lagrange function, $F_{k}=-2\zeta_{k}\omega_{k}{\dot{r}}_{k}$ is the dissipative force, $\omega_{k}$ is the natural frequency of the oscillator, and $Q_{k}=V_{k}/R_{L}$ is the output current. The dynamic equations can be obtained as

(24) $$\begin{array}{c} {\ddot{r}}_{1}+{\omega_{1}}^{2}r_{1}+2\omega_{n1}\zeta_{1}{\dot{r}}_{1}-\theta_{1}V_{1}+g_{m1}+m_{t1}\left(g+{\ddot{z}}_{b}\right)=0,\\ \theta_{1}{\dot{r}}_{1}+\frac{1}{2}C_{p1}{\dot{V}}_{1}+\frac{V_{1}}{R}=0,\\ {\ddot{r}}_{2}+{\omega_{2}}^{2}r_{2}+2\omega_{2}\zeta_{2}{\dot{r}}_{2}-\theta_{2}V_{1}+g_{m2}+m_{t2}\left(g+{\ddot{z}}_{b}\right)=0,\\ \theta_{2}{\dot{r}}_{2}+\frac{1}{2}C_{p2}{\dot{V}}_{2}+\frac{V_{2}}{R}=0, \end{array}$$

where

(25) $$\begin{array}{c} {\omega_{1}}^{2}=C_{s}I_{s1}\int_{0}^{L_{s1}}{{\phi^{\prime \prime }}_{2}}^{2}dx+C_{11}I_{p1}\int_{0}^{L_{p1}}{{\phi^{\prime \prime }}_{1}}^{2}dx,\\ {\omega_{2}}^{2}=C_{s}I_{s2}\int_{0}^{L_{s2}}{{\phi^{\prime \prime }}_{2}}^{2}dx+C_{11}I_{p2}\int_{0}^{L_{p2}}{{\phi^{\prime \prime }}_{2}}^{2}dx,\\ \theta_{1}=\frac{1}{2}e_{31}h_{p}b_{p1}\left(h_{p}+h_{s}\right)\int_{0}^{L_{p1}}{\phi^{\prime \prime }}_{1}dx,\\ \theta_{2}=\frac{1}{2}e_{31}h_{p}b_{p2}\left(h_{p}+h_{s}\right)\int_{0}^{L_{p2}}{\phi^{\prime \prime }}_{2}dx,\\ m_{t1}=\rho_{s}A_{s1}\int_{0}^{L_{s1}}\phi_{1}dx+2\rho_{p}A_{p1}\int_{0}^{L_{P1}}\phi_{1}dx+m\phi_{1}\left(L_{s1}\right),\\ m_{t2}=\rho_{s}A_{s2}\int_{0}^{L_{s2}}\phi_{2}dx+2\rho_{p}A_{p2}\int_{0}^{L_{p2}}\phi_{2}dx+m\phi_{2}\left(L_{s2}\right), \end{array}$$

where $\phi_{k}\left(k=1,2\right)$ are the normalized mode shapes of the oscillators and $g_{mk}=\partial U_{m}/\partial r_{k}$ are the magnetic forces. For the base motion, we set

(26) $${\ddot{z}}_{b}=A\cos2\pi ft,$$

where *A* is the amplitude of the acceleration of the basic motion and *f* is the excitation frequency.

## 3. Static Characteristics of the PEH

This section examines the impact of magnet spacing on the static equilibrium state and potential energy distribution within the PEH. Unless stated otherwise, all subsequent analyses will utilize the parameter values provided in Table 1.

**Figure 3:**
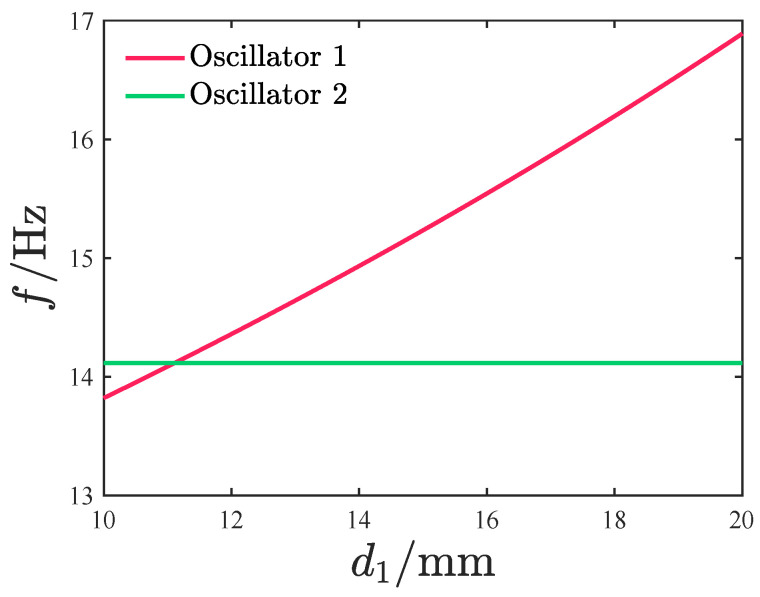
The natural frequency of the oscillator varies with $d_{1}$.

It is noted that the variation in the moving-magnet clearance $d_{1}$ is determined by the length of the oscillators. Specifically, we maintain the length of oscillator 2 as constant while exclusively altering the length of oscillator 1. Since any change in the oscillator’s length directly affects its linear natural frequency, Figure 3 illustrates the corresponding variation in the oscillator’s natural frequency with respect to $d_{1}$. As shown in the figure, an increase in $d_{1}$ reduces the length of oscillator 1, thereby enhancing its rigidity and increasing its natural frequency proportionally. When $d_{1}$ is relatively small, the natural frequencies of the two oscillators are closely matched. For the specific case $d_{1}=16\;\mathrm{mm}$, the natural frequencies of oscillators 1 and 2 are measured as 14.11 Hz and 15.54 Hz, respectively.

**Figure 4:**
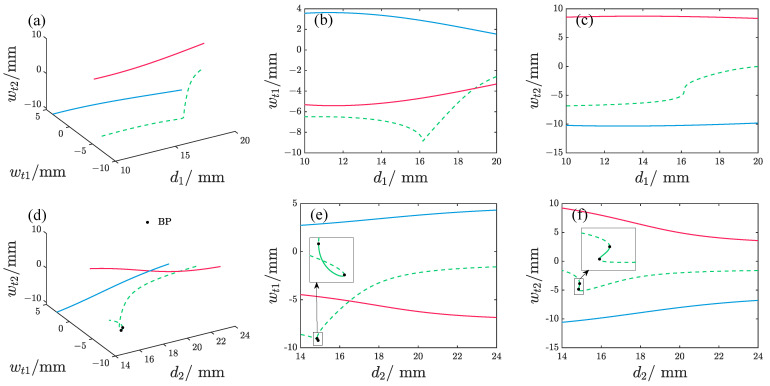
The effect of magnet spacing on the static equilibrium position of the repulsive-type PEH, where different colors represent different solution branches, the solid line denotes the stable solution, and the dash line denotes the unstable solution; BP denotes the bifurcation point. (**b**,**c**) represent the orthogonal projections of (**a**) onto the $d_{1}-w_{t1}$ plane and $d_{1}-w_{t2}$ plane, respectively. (**e**,**f**) represent the orthogonal projections of (**d**) onto the $d_{2}-w_{t1}$ plane and $d_{2}-w_{t2}$ plane, respectively.

**Figure 5:**
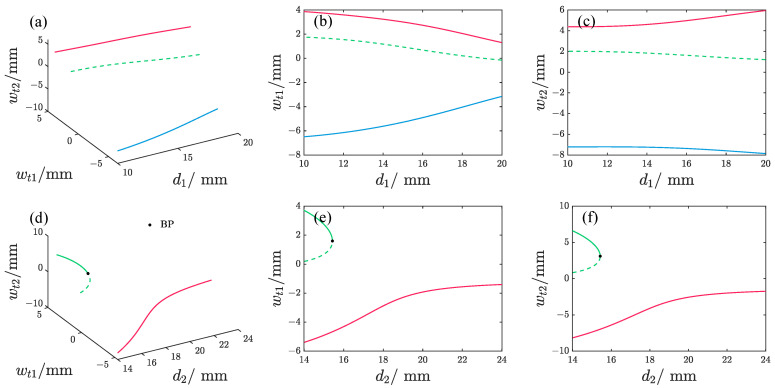
The effect of magnet spacing on the static equilibrium position of the attractive-type PEH, where different colors represent different solution branches, the solid line denotes the stable solution, and the dash line denotes the unstable solution; BP denotes the bifurcation point. (**b**,**c**) represent the orthogonal projections of (**a**) onto the $d_{1}-w_{t1}$ plane and $d_{1}-w_{t2}$ plane, respectively. (**e**,**f**) represent the orthogonal projections of (**d**) onto the $d_{2}-w_{t1}$ plane and $d_{2}-w_{t2}$ plane, respectively.

Figure 4 and Figure 5 demonstrate the impact of varying magnet spacing on the static equilibrium positions of the repulsive-type and attractive-type PEHs, respectively. To facilitate clear visualization of the analysis results, the displacements of the free ends of the oscillators $w_{t1}$ and $w_{t2}$ are used to characterize the static equilibrium states. As observed, changes in $d_{1}$ lead to both the repulsive-type and attractive-type PEHs exhibiting three static equilibrium positions, comprising two stable and one unstable configuration. When $d_{2}$ is varied, the repulsive-type PEH maintains two stable equilibrium points and one unstable point for most $d_{2}$ values. However, near $d_{2}$ = 14.9 mm, five equilibrium points emerge, with three of them being stable. In contrast, the attractive-type PEH exhibits three equilibrium points at small $d_{2}$ but transitions to a single stable equilibrium as $d_{2}$ increases.

**Figure 6:**
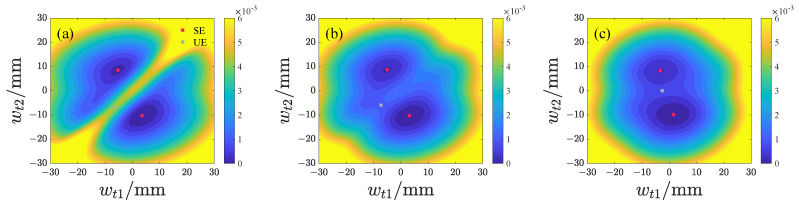
Potential energy distribution of the repulsive-type PEH at different $d_{1}$ values, where SE denotes the stable equilibrium and UE denotes the unstable equilibrium: (**a**) $d_{1}$ = 10 mm. (**b**) $d_{1}$ = 15 mm. (**c**) $d_{1}$ = 20 mm.

**Figure 7:**
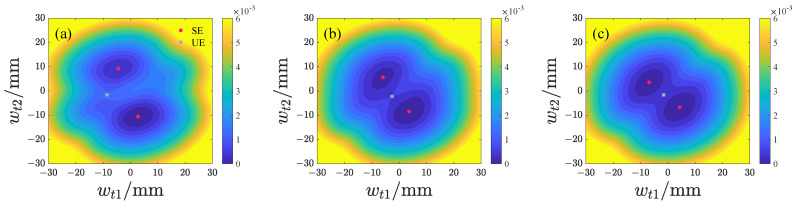
Potential energy distribution of the repulsive-type PEH at different $d_{2}$ values, where SE denotes the stable equilibrium and UE denotes the unstable equilibrium: (**a**) $d_{2}$ = 14 mm. (**b**) $d_{2}$ = 19 mm. (**c**) $d_{2}$ = 24 mm.

**Figure 8:**
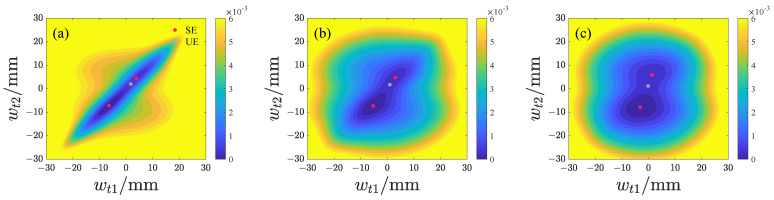
Potential energy distribution of the attractive-type PEH at different $d_{1}$ values, where SE denotes the stable equilibrium and UE denotes the unstable equilibrium: (**a**) $d_{1}$ = 10 mm. (**b**) $d_{1}$ = 15 mm. (**c**) $d_{1}$ = 20 mm.

**Figure 9:**
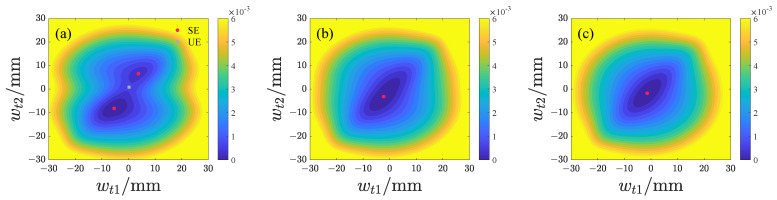
Potential energy distribution of the attractive-type PEH at different $d_{2}$ values, where SE denotes the stable equilibrium and UE denotes the unstable equilibrium: (**a**) $d_{2}$ = 14 mm. (**b**) $d_{2}$ = 19 mm. (**c**) $d_{2}$ = 24 mm.

We subsequently analyze the effects of $d_{1}$ and $d_{2}$ on the potential energy distribution of both repulsive-type and attractive-type PEHs, with the results presented in Figure 6, Figure 7, Figure 8 and Figure 9 and the potential energy given by Equation (18). In this analysis, the lowest point on the potential energy surface is designated as the zero-potential-energy reference position. It is evident that the repulsive-type PEH exhibits a dual potential well structure, where the equilibrium points are distributed along the upper-left and lower-right directions. As $d_{1}$ and $d_{2}$ increase, the unstable equilibrium point progressively shifts from the left side toward the region between the two stable equilibrium points. Concurrently, the area of the potential well gradually expands. For the attractive-type PEH, increasing $d_{1}$ results in a gradual expansion of both the potential well’s width and area. Conversely, as $d_{2}$ increases, the system undergoes a transition from a dual potential well to a single potential well, with the potential well area gradually decreasing.

The height of the potential barrier between the two potential wells can significantly influence the performance of PEHs. A lower barrier height makes it easier for the system to overcome the intermediate barrier, potentially leading to large vibration responses. Consequently, we analyzed the effects of $d_{1}$ and $d_{2}$ on the intermediate barrier height for both the repulsive-type and attractive-type PEHs.

**Figure 10:**
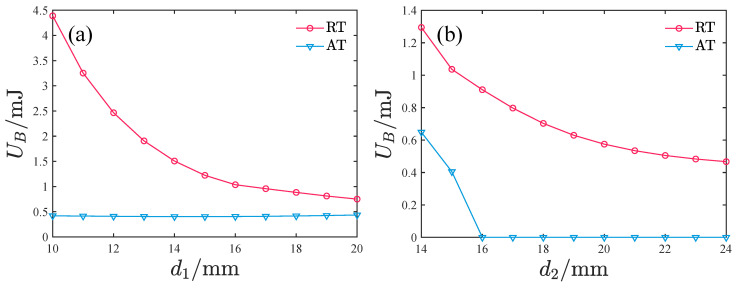
Effect of $d_{1}$ and $d_{2}$ on the potential barrier height, where RT denotes the repulsive-type PEH and AT denotes the attractive-type PEH: (**a**) Effect of $d_{1}$. (**b**) Effect of $d_{2}$.

As shown in Figure 10, the barrier height of the repulsive-type PEH decreases with increasing $d_{1}$ and $d_{2}$. In contrast, the barrier height of the attractive-type PEH gradually increases with $d_{1}$ but decreases rapidly as $d_{2}$ rises. At $d_{2}$ = 16 mm, the barrier disappears entirely, transitioning the system to a single potential well configuration. Additionally, it is noteworthy that the barrier height of the attractive-type PEH is consistently much lower than that of the repulsive-type PEH under comparable conditions.

## 4. Dynamic Characteristics and Performances of the PEHs

This section presents simulation studies of the PEH’s dynamic equations. The 4th-order Runge–Kutta method is applied to solve dynamic equations to investigate the effects of acceleration amplitude and magnet spacing on the system’s dynamic behavior and energy harvesting performance.

### 4.1. Dynamic Characteristics and Performances of the Repulsive-Type PEH

**Figure 11:**
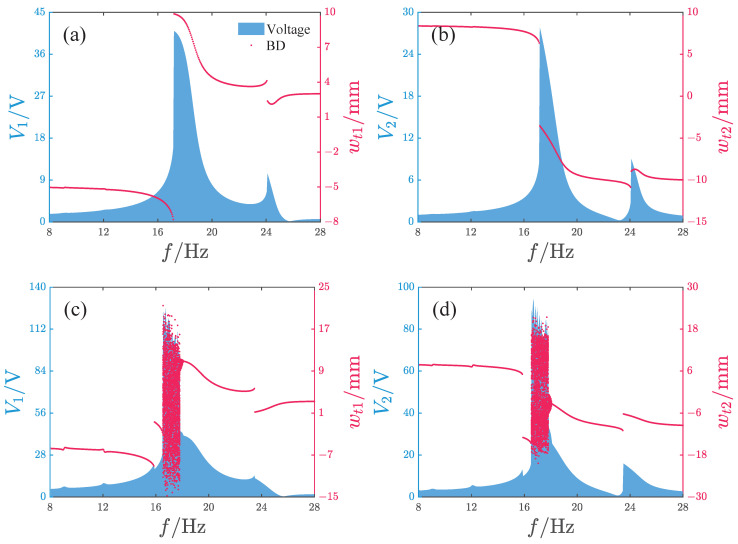
Output characteristics of the repulsive-type PEH, where Voltage denotes the output voltage of the PEH, BD denotes the bifurcation diagram, and $V_{1}$ and $V_{2}$ are the output voltages of oscillator 1 and 2, respectively: (**a**,**b**) $A=2.828\;m/s^{2}$. (**c**,**d**) $A=8.485\;m/s^{2}$.

Figure 11 shows the output characteristics of the repulsive-type PEH. We analyze two acceleration amplitudes: $A=2.828\;m/s^{2}$ (root mean square (RMS) value of 2 $\;m/s^{2}$) and $A=8.485\;m/s^{2}$ (RMS value of 6 $\;m/s^{2}$). During the calculation, the excitation frequency is gradually increased from 8 Hz to 28 Hz. $V_{1}$ and $V_{2}$ represent the voltage outputs of oscillator 1 and oscillator 2, respectively.

When the acceleration amplitude $A=2.828\;m/s^{2}$, as the excitation frequency increases, the outputs of $V_{1}$ and $V_{2}$ both exhibit two peaks. These peaks are located near 17.2 Hz and 24.1 Hz, respectively. At this time, the maximum values of $V_{1}$ and $V_{2}$ are 40.9 V and 27.8 V, respectively. From bifurcation diagram analysis, it is known that the system’s response exhibits period-1 motion across the entire frequency range and that a jump occurs at each of the two peak positions.

When the acceleration amplitude is increased to $A=8.485\;m/s^{2}$, the system’s voltage output exhibits a large peak within the frequency range of 16.6–17.8 Hz, with the maximum values of $V_{1}$ and $V_{2}$ being 123 V and 91 V, respectively. Notably, the response near this peak exhibits non-periodic behavior. In addition, the system’s voltage output shows four smaller peaks at 9 Hz, 12 Hz, 15.8 Hz, and 23.4 Hz, respectively. The response amplitude jumps at 15.8 Hz, 16.6 Hz, 17.8 Hz, and 23.4 Hz.

Furthermore, we observe that across different acceleration values, the peak response frequencies are all greater than the oscillators’ natural frequencies. This phenomenon indicates that the introduction of magnetic forces increases the system’s resonance frequency.

**Figure 12:**
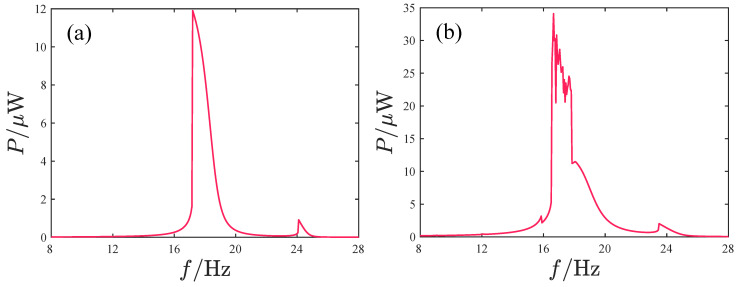
Variation in the output power with the excitation frequency corresponding to different acceleration amplitudes: (**a**) A=2.828 $m/s^{2}$. (**b**) A=8.485 $m/s^{2}$.

Since the voltage amplitude cannot fully reflect the system’s harvesting performance, Figure 12 presents the output power variation curves with frequency, where the output power is defined as the average power of the system’s response over 100 excitation cycles at each frequency.

(27) $$P=\frac{1}{100T}\int_{0}^{100T}\left({V_{1}}^{2}+{V_{2}}^{2}\right)/R_{L}dt.$$

The trends in the power curves and voltage outputs are similar. However, we also notice that when the acceleration amplitude A=8.485 $m/s^{2}$, the voltage peaks of $V_{1}$ and $V_{2}$ are approximately three times those at A=2.828 $m/s^{2}$. Yet, from the perspective of power output, their peak power ratios are still less than three times greater. This indicates that the true harvesting performance in the non-periodic response regions is not as prominent as its voltage peaks suggest.

**Figure 13:**
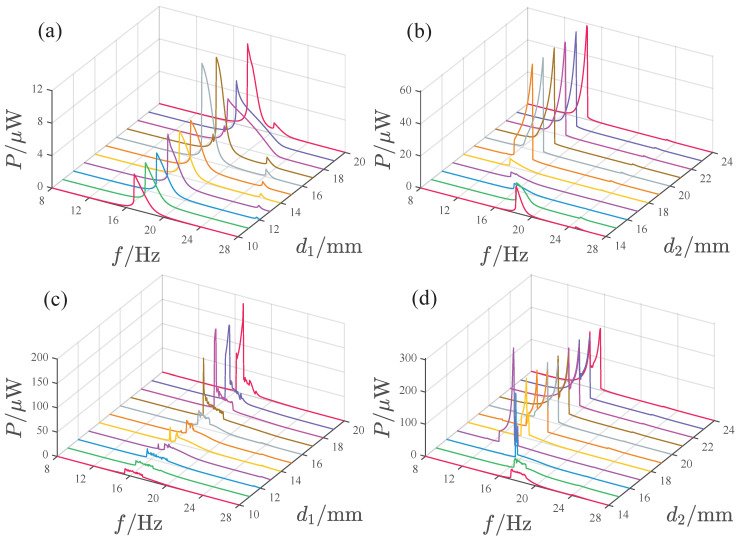
Effect of $d_{1}$ and $d_{2}$ on the output power of the PEH: (**a**,**b**) $A=2.828\;m/s^{2}$. (**c**,**d**) $A=8.485\;m/s^{2}$.

Figure 13 illustrates the influence of magnet spacing $d_{1}$ and $d_{2}$ on the power output. At *A* = 2.828 $m/s^{2}$, the system’s peak power gradually increases with $d_{1}$ within the $d_{1}$ = 10–15 mm range, significantly increases at $d_{1}=16\;\mathrm{mm}$, then suddenly decreases at $d_{1}=18\;\mathrm{mm}$ and 19 mm and increases again at $d_{1}=20\;\mathrm{mm}$. The maximum peak output is approximately 15μW, occurring at $d_{1}=20\;\mathrm{mm}$. Besides the main peak, the system also exhibits a smaller peak with a higher frequency. When $d_{1}=18$ and 19 mm, these two peaks merge into a broadband peak. The variation in $d_{1}$ has little effect on the frequency of the main peak, which remains concentrated in the 16–20 Hz range. The frequency of the smaller peak gradually decreases with increasing $d_{1}$. When $d_{2}$ is in the 14–18 mm range, both the system’s peak power and its corresponding frequency gradually decrease with increasing $d_{2}$. When $d_{2}\geq 19\;\mathrm{mm}$, the system response shows a significant jump and the peak output after the jump approaches 60μW. At this point, the peak power and frequency are essentially unchanged with further variation in $d_{2}$. Under the acceleration condition $A=8.485\;m/s^{2}$, the response frequency band is mainly located in the 16–20 Hz range. When $d_{1}\leq 16\;\mathrm{mm}$, the system response peak is small. As $d_{1}$ increases, the response peak suddenly increases at $d_{1}=17\;\mathrm{mm}$. Within the $d_{1}$ = 17–20 mm range, the response peak gradually increases with $d_{1}$, approaching 200μW at $d_{1}=20\;\mathrm{mm}$. Regarding the influence of $d_{2}$, when $d_{2}\leq 15\;\mathrm{mm}$, the response peak is small. At the $d_{2}=16\;\mathrm{mm}$ position, the peak response suddenly increases. At $d_{2}$ = 17 mm, the response peak with increased frequency reaches its maximum value of approximately 300μW. After $d_{2}\geq 18\;\mathrm{mm}$, the response exhibits a double peak, with the response peak frequency band mainly concentrated in the 16–20 Hz range and the response peak stabilizing around 200μW.

**Figure 14:**
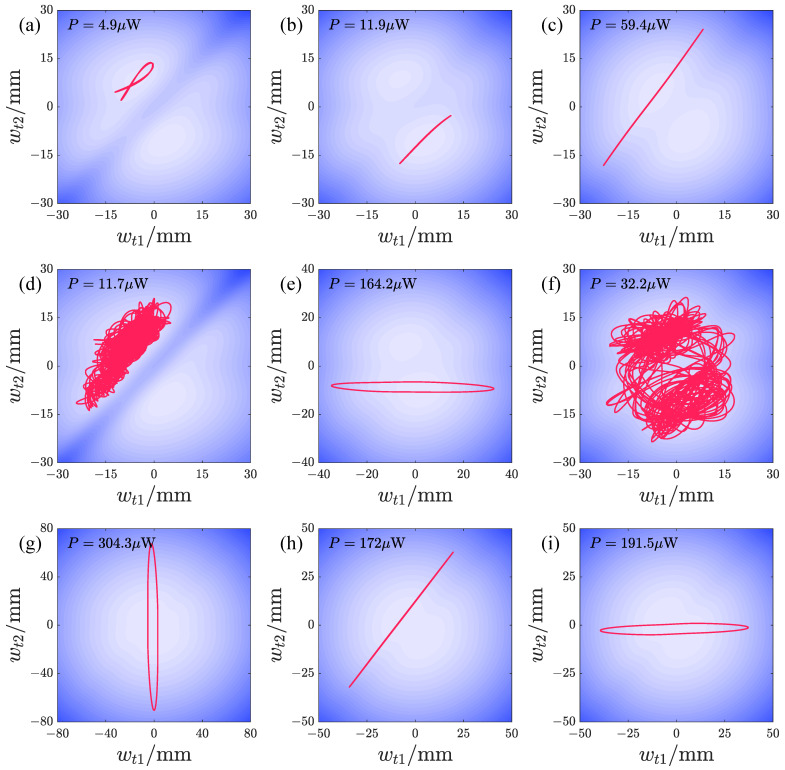
The typical peak responses of the repulsive-type PEH: (**a**) *A* = 2 $\;m/s^{2}$, $d_{1}$ = 16 mm, $d_{2}$ = 12 mm, *f* = 16.95 Hz. (**b**) *A* = 2 $\;m/s^{2}$, $d_{1}$ = 16 mm, $d_{2}$ = 15 mm, *f* = 17.2 Hz. (**c**) *A* = 2 $\;m/s^{2}$, $d_{1}$ = 16 mm, $d_{2}$ = 20 mm, *f* = 14.3 Hz. (**d**) *A* = 6 $\;m/s^{2}$, $d_{1}$ = 10 mm, $d_{2}$ = 15 mm, *f* = 15.45 Hz. (**e**) *A* = 6 $\;m/s^{2}$, $d_{1}$ = 18 mm, $d_{2}$ = 15 mm, *f* = 16.2 Hz. (**f**) *A* = 6 $\;m/s^{2}$, $d_{1}$ = 16 mm, $d_{2}$ = 14 mm, *f* = 17.45 Hz. (**g**) *A* = 6 $\;m/s^{2}$, $d_{1}$ = 16 mm, $d_{2}$ = 17 mm, *f* = 14.15 Hz. (**h**) *A* = 6 $\;m/s^{2}$, $d_{1}$ = 16 mm, $d_{2}$ = 24 mm, *f* = 14.4 Hz. (**i**) *A* = 6 $\;m/s^{2}$, $d_{1}$ = 16 mm, $d_{2}$ = 24 mm, *f* = 15.55 Hz.

We also present the typical peak responses of the repulsive-type PEH. Figure 14a,b, and c show responses at lower acceleration amplitudes, with in-phase or approximately in-phase responses within a single potential well. When the acceleration amplitude is larger, varying $d_{1}$ can lead to non-periodic responses (see Figure 14d), as well as periodic responses with large-amplitude vibration of oscillator 1 and small-amplitude vibration of oscillator 2 (see Figure 14e). These are still confined within one potential well and do not cross the intermediate barrier. As $d_{2}$ varies, the response forms become more diverse. Figure 14f shows a non-periodic response crossing the double potential well, but the vibration is primarily within one well, with relatively few trajectories crossing the intermediate barrier. Figure 14g,i show periodic responses crossing the dual potential well, characterized by the large-amplitude vibration of one oscillator and small-amplitude vibration of the other. Figure 14h shows large-amplitude vibration within a single potential well, with both oscillators vibrating in-phase and having relatively large amplitudes.

**Figure 15:**
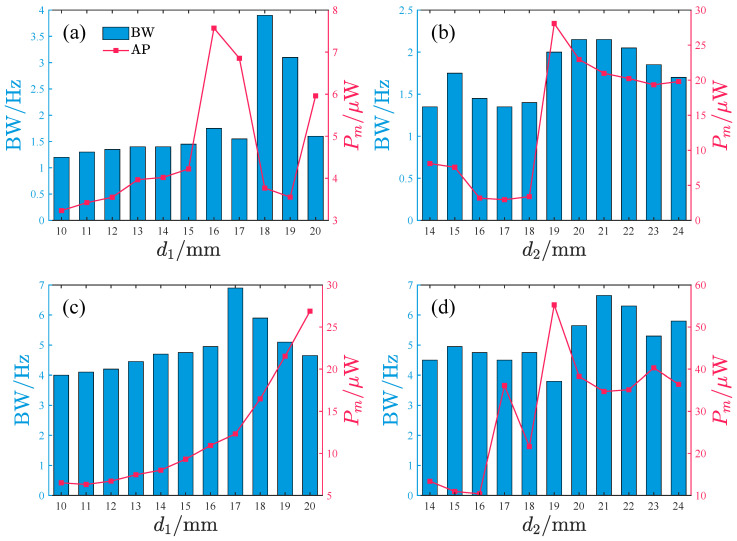
The bandwidth with power ≥2μW and the average power within the bandwidth vary with $d_{1}$ and $d_{2}$, where BW denotes the bandwidth and $P_{m}$ denotes the average power within the bandwidth: (**a**,**b**) *A* = 2.828$\;m/s^{2}$. (**c**,**d**) *A* = 8.485$\;m/s^{2}$.

To investigate the effects of $d_{1}$ and $d_{2}$ on the harvesting performance, we specifically examine the operational bandwidth where output power exceeds 2μW, along with the average power characteristics within this bandwidth. The results, as depicted in Figure 15, demonstrate the systematic influence of $d_{1}$ and $d_{2}$ variations.

When the acceleration amplitude is small, the bandwidth initially increases and subsequently decreases with increasing $d_{1}$, with optimal bandwidth observed at $d_{1}$ = 18–19 mm. The average power exhibits a non-monotonic trend (increase → decrease → increase), peaking at $d_{1}=16,17$, and 20 mm. For $d_{2}$, the bandwidth remains relatively stable, whereas the average power shows a sharp increase at $d_{2}$ = 19 mm, maintaining high values within $d_{2}$ = 19–24 mm while gradually declining with further increases in $d_{2}$.

Under large acceleration amplitudes, the bandwidth increases slowly with $d_{1}$ in the range $d_{1}$ = 10–16 mm, followed by a sudden surge and rapid decay at $d_{1}$ = 17–20 mm. The average power monotonically increases with $d_{1}$, reaching about 27μW at $d_{1}=20\;\mathrm{mm}$. The $d_{2}$ influence is more complex: within $d_{2}$ = 20–24 mm, the system simultaneously achieves favorable bandwidth and average power, with the bandwidth gradually decreasing and the output power showing mild fluctuations as $d_{2}$ increases.

Larger $d_{1}$ and $d_{2}$ values generally enhance bandwidth and average power for the repulsive-type PEH. However, performance degradation occurs when $d_{2}>19\;\mathrm{mm}$. Regarding the peak response frequency, the influence of $d_{1}$ on the primary peak frequency band is negligible, while the frequency band of small-amplitude response decreases with increasing $d_{2}$ and the frequency band of large-amplitude response remains unaffected by changes in $d_{2}$.

### 4.2. Dynamic Characteristics and Performances of the Attractive-Type PEH

**Figure 16:**
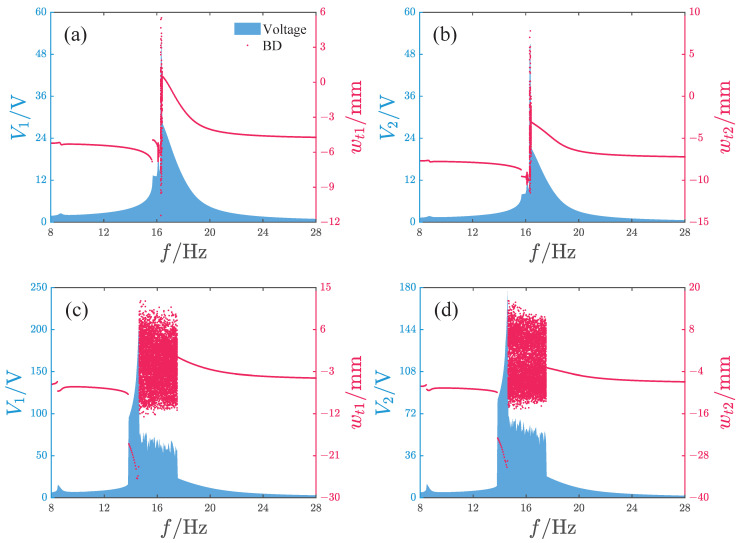
Output characteristics of the attractive-type PEH, where Voltage denotes the output voltage of the PEH, BD denotes the bifurcation diagram, and $V_{1}$ and $V_{2}$ are the output voltages of oscillator 1 and 2, respectively: (**a**,**b**) *A* = 2.828$\;m/s^{2}$. (**c**,**d**) *A* = 8.485$\;m/s^{2}$.

**Figure 17:**
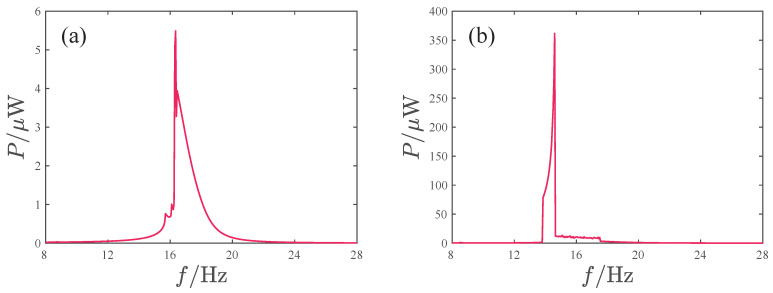
Variation in the output power with the excitation frequency corresponding to different acceleration amplitudes: (**a**) *A* = 2.828$\;m/s^{2}$. (**b**) *A* = 8.485$\;m/s^{2}$.

Figure 16 and Figure 17 show the response characteristics and voltage/power output of the attractive-type PEH under different acceleration amplitudes. When the acceleration is small, the system response mainly exhibits period-1 motion, and as the frequency increases, a significant peak appears, with the main peak frequency band concentrated in the range of 16–18 Hz. Although the maximum output voltages of the two oscillators exceed 45 V, such high-voltage outputs exist only in a very narrow frequency interval near the peak. For other frequencies, the voltage output of oscillator 1 is below 30 V and the voltage output of oscillator 2 is less than 22 V. When the acceleration amplitude increases, the system output voltage increases significantly, with the maximum voltages of oscillator 1 and 2 reaching approximately 204 V and 177 V, respectively, and the peak frequency being at 14.6 Hz. The system response with a larger amplitude is mainly concentrated in the frequency band 13.7–17.5 Hz, where the response is primarily non-periodic; only near the peak is there a periodic response with large amplitude. The system power output is similar to the voltage output, with the output power peaks corresponding to the two acceleration amplitudes being 5.5 μW and 361.6 μW and the peak frequencies being at 16.35 Hz and 14.6 Hz, respectively.

**Figure 18:**
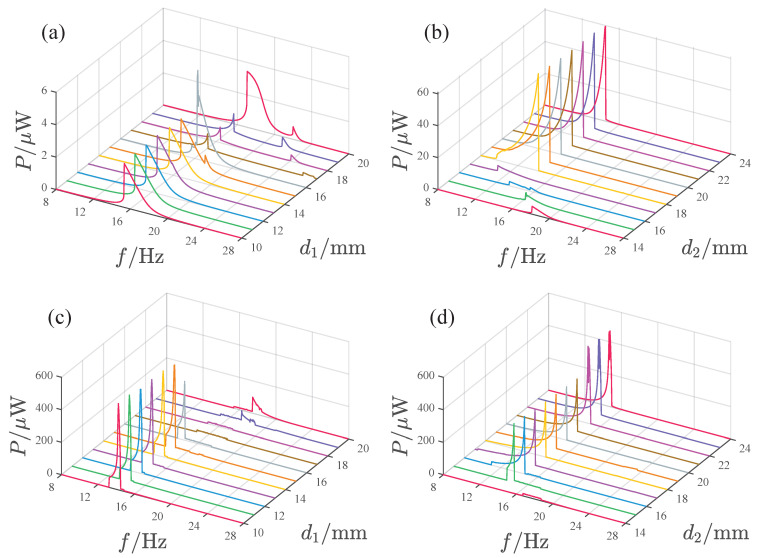
Effect of $d_{1}$ and $d_{2}$ on the output power of the PEH: (**a**,**b**) *A* = 2.828$\;m/s^{2}$. (**c**,**d**) *A* = 8.485$\;m/s^{2}$.

Figure 18 shows the influence of $d_{1}$ and $d_{2}$ on the power output of the attractive-type PEH. At lower acceleration amplitudes, when $d_{1}$ is in the range of 10–16 mm, the system response shows a main peak as a function of frequency, with a peak frequency band of 14.5–18 Hz, and the system’s power output is relatively close. When $d_{1}$ is in the 17–19 mm range, the response peak significantly decreases and the bandwidth narrows. When $d_{1}$ is 20 mm, the response increases again. The variation in power output with $d_{2}$ is as follows: when $d_{2}$ is in the 14–17 mm range, the system’s power output is very small, and the peak frequency gradually decreases with increasing $d_{2}$. When $d_{2}$ is in the 18–24 mm range, the system has a larger power output, with the peak power basically being stable at 60μW, but the range of the peak frequency band gradually narrows with increasing $d_{2}$.

Under high acceleration amplitude, the PEH exhibits higher power output in the $d_{1}$ range of 10–16 mm. For $d_{1}$ values from 10 to 15 mm, the output power remains nearly constant as $d_{1}$ increases, with a peak output of approximately 530μW. The output decreases slightly at $d_{1}$ = 16 mm. The peak frequency band in this range is essentially located at 13–15 Hz. After $d_{1}\geq$ 17 mm, the output power of the PEH decreases significantly, and both the peak power and frequency gradually increase with $d_{1}$.

The variation in power output with $d_{2}$ is as follows: The output is very small at $d_{2}$ = 14 mm. The peak power output suddenly increases at $d_{2}$ = 15 mm and remains around 350μW in the 15–21 mm interval. At $d_{2}$ = 22 mm, the peak power output increases again and stays around 465μW in the 22–24 mm interval. Within the $d_{2}$ range of 15–24 mm, the peak frequency band remains essentially unchanged at 12–16 Hz.

**Figure 19:**
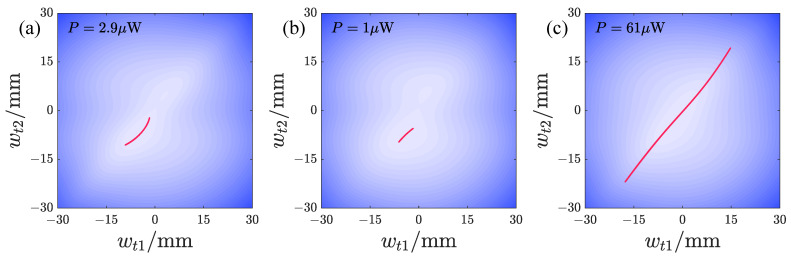

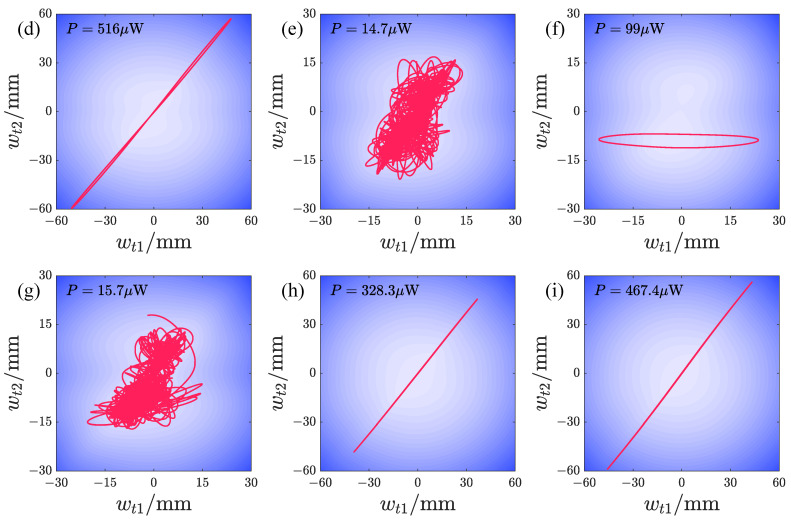
The typical peak responses of the attractive-type PEH: (**a**) *A* = 2.828$\;m/s^{2}$, $d_{1}$ = 15 mm, $d_{2}$ = 15 mm, *f* = 15.7 Hz; (**b**) *A* = 2.828$\;m/s^{2}$, $d_{1}$ = 18 mm, $d_{2}$ = 15 mm, *f* = 16.45 Hz; (**c**) *A* = 2.828$\;m/s^{2}$, $d_{1}$ = 16 mm, $d_{2}$ = 18 mm, *f* = 14.2 Hz; (**d**) *A* = 8.485$\;m/s^{2}$, $d_{1}$ = 15 mm, $d_{2}$ = 15 mm, *f* = 14.65 Hz; (**e**) *A* = 8.485$\;m/s^{2}$, $d_{1}$ = 17 mm, $d_{2}$ = 15 mm, *f* = 14.85 Hz; (**f**) *A* = 8.485$\;m/s^{2}$, $d_{1}$ = 20 mm, $d_{2}$ = 15 mm, *f* = 17.45 Hz; (**g**) *A* = 8.485$\;m/s^{2}$, $d_{1}$ = 16 mm, $d_{2}$ = 14 mm, *f* = 16.75 Hz; (**h**) *A* = 8.485$\;m/s^{2}$, $d_{1}$ = 16 mm, $d_{2}$ = 20 mm, *f* = 14.6 Hz; (**i**) *A* = 8.485$\;m/s^{2}$, $d_{1}$ = 16 mm, $d_{2}$ = 24 mm, *f* = 14.75 Hz.

Figure 19 shows the typical peak responses of the attractive-type PEH. Figure 19a,b,d–g correspond to the double-well state. Figure 19a,b are small-amplitude in-well vibrations at smaller accelerations. Figure 19c is a large-amplitude vibration within a single well at a smaller acceleration and larger $d_{2}$. Figure 19d shows a large-amplitude vibration response at a larger acceleration amplitude and smaller $d_{1}$, crossing the intermediate potential barrier. Figure 19e,g are non-periodic responses crossing the intermediate potential barrier, but their responses are concentrated within the double-well region. Figure 19h,i are large-amplitude vibrations within a single well. Figure 19f shows a response with a large vibration amplitude of oscillator 1 and a small amplitude of oscillator 2. It can be seen that the periodic responses of the attractive-type PEH are mainly in-phase, consistent with the potential energy distribution, which is conducive to crossing the intermediate potential barrier and forming large-amplitude vibration responses. For the large-amplitude responses shown in Figure 19d,h,i, the amplitudes of both oscillators are large, resulting in good output power. In contrast, the non-periodic responses are largely confined to the potential well, so their output power is lower.

**Figure 20:**
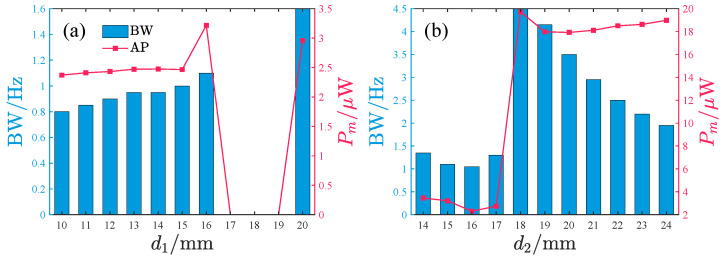

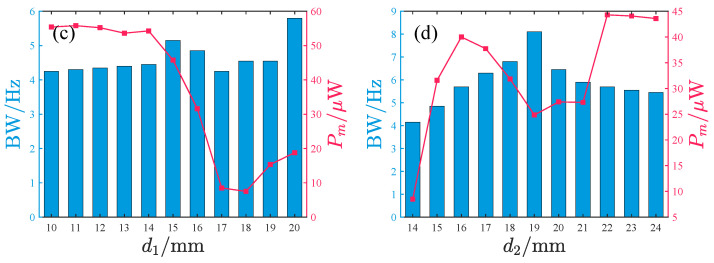
The bandwidth with power ≥2μW and the average power within the bandwidth vary with $d_{1}$ and $d_{2}$, where BW denotes the bandwidth and $P_{m}$ denotes the average power within the bandwidth: (**a**,**b**) *A* = 2.828$\;m/s^{2}$. (**c**,**d**) *A* = 8.485$\;m/s^{2}$.

Figure 20 shows the influence of $d_{1}$ and $d_{2}$ on the bandwidth (≥2μW) and the average power within this bandwidth of the attractive-type PEH. Corresponding to given acceleration amplitudes, when $d_{1}$ is in the 17–19 mm range, the average power output significantly decreases, whereas when $d_{1}$ is smaller, the system exhibits better bandwidth characteristics and average power performance. Under low-acceleration-amplitude conditions, a larger $d_{2}$ value corresponds to a larger bandwidth and higher average power. However, within this $d_{2}$ range, the bandwidth shows a rapid decay trend while the average power remains basically stable in the 18–20μW interval. When the acceleration amplitude is large, the bandwidth first increases and then decreases with increasing $d_{2}$; the average power performs better in the $d_{2}$ range of 22–24 mm, but within this interval, both bandwidth and average power decrease slowly with increasing $d_{2}$.

Overall, for the attractive-type PEH, a smaller $d_{1}$ and a larger $d_{2}$ are more likely to achieve a larger bandwidth and average power. However, similar to the repulsive-type PEH, within the better-performing interval, the PEH’s performance gradually decreases with increasing $d_{2}$. From the perspective of the main peak frequency band of the response, the peak frequency of the small-amplitude response gradually decreases with increasing $d_{2}$ and gradually increases with increasing $d_{1}$, while the peak frequency of the large-amplitude response is basically unaffected by $d_{1}$ and $d_{2}$.

### 4.3. Performance Comparison Between the Repulsive-Type and Attractive-Type PEHs

Considering both bandwidth and average power, we define the performance of the PEH as

(28) $$\mathrm{Perf}.=\mathrm{BW}\cdot P_{m}.$$

**Figure 21:**
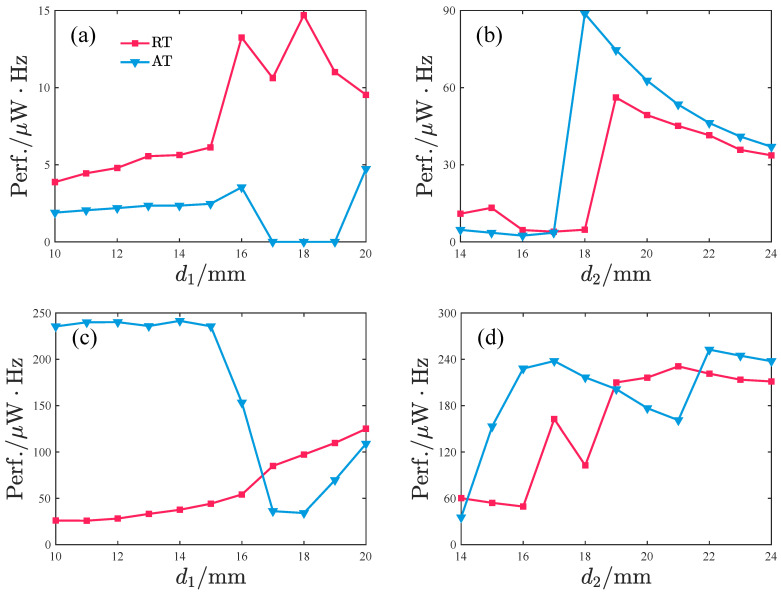
Comparison of the performance between the repulsive-type and attractive-type PEHs, where RT denotes the repulsive-type PEH and AT denotes the attractive-type PEH: (**a**,**b**) $A=2.828\;m/s^{2}$; (**c**,**d**) $A=8.485\;m/s^{2}$.

Figure 21 presents a comparison of the performance between the repulsive-type and attractive-type PEHs. In the parameter regions corresponding to the poorer-performing parts of Figure 21a,b, the repulsive-type PEH exhibits superior performance. This is because, under these parameters, the response of both PEHs is in-phase, small-amplitude vibration within the potential well, and the repulsive-type PEH’s potential well is wider in that direction. In other parameter intervals, the attractive-type PEH outperforms the repulsive-type PEH. This is because the attractive-type PEH’s response is in-phase vibration, consistent with the direction of its potential well distribution, making it easier to overcome the barrier of the intermediate potential well and form large-amplitude vibrations, with both oscillators simultaneously possessing large amplitudes. The repulsive-type PEH’s response form and the direction of its potential well distribution differ significantly, which is unfavorable for the emergence of large-amplitude vibration responses. Moreover, the larger-power peak responses primarily consist of one oscillator vibrating with large amplitude and the other with small amplitude, resulting in relatively smaller output power.

**Figure 22:**
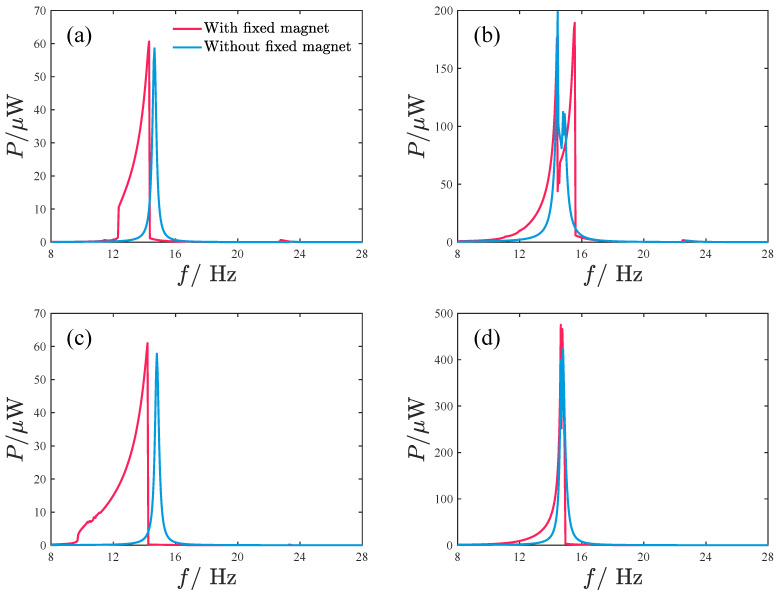
Comparison of the responses between the PEHs with and without fixed magnets: (**a**) the repulsive-type PEH: $d_{1}$ = 16 mm, $d_{2}$ = 19 mm, $A=2.828\;m/s^{2}$; (**b**) the repulsive-type PEH: $d_{1}$ = 16 mm, $d_{2}$ = 21 mm, $A=8.485\;m/s^{2}$; (**c**) $d_{1}$ = 16 mm, $d_{2}$ = 18 mm, $A=2.828\;m/s^{2}$; (**d**) $d_{1}$ = 16 mm, $d_{2}$ = 22 mm, $A=8.485\;m/s^{2}$.

We also conduct a comparison of the PEH proposed in this paper with the typical dual-oscillator coupled PEH without fixed magnets, as shown in Figure 22. In the analysis, the oscillator length and moving-magnet spacing $d_{1}$ are kept constant, and the responses of the repulsive-type and attractive-type PEHs without fixed magnets are calculated. The responses for the PEH with a fixed magnet are taken from the optimal values in Figure 21b,d. Clearly, the PEH with a fixed magnet exhibits superior response performance. Table 2 provides more detailed performance data, showing that the inclusion of the fixed magnet significantly enhances both the system bandwidth and average output power. In particular, under smaller excitation acceleration amplitudes, the performance improvement reaches 128% for the repulsive type and 364% for the attractive type. Furthermore, it is observed that the PEH with fixed magnets has a lower bandwidth frequency, indicating that the proposed design is more suitable for energy harvesting under low-frequency and small-amplitude excitation conditions.

## 5. Experimental Verification

**Figure 23:**
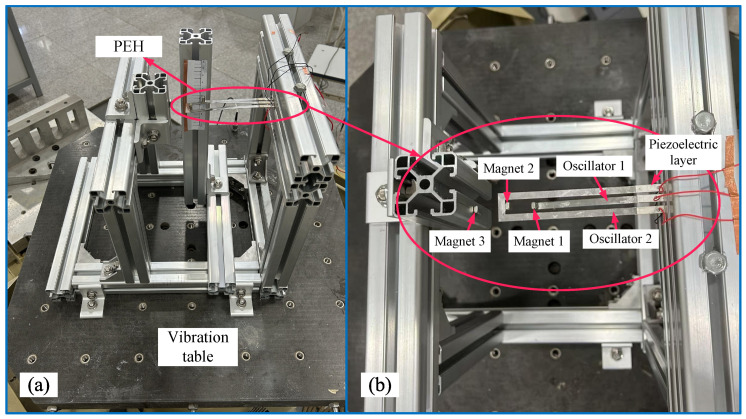
Experimental prototype of the dual-oscillator PEH with external magnet: (**a**) Experimental prototype. (**b**) Local enlargement of the dual-oscillator PEH with external magnet.

To validate the numerical simulations, as shown in Figure 23, we fabricate an experimental prototype of the dual-oscillator PEH. The outer beam length is set to 108 mm and the inner beam length to 92 mm. The distance between the external fixed magnet and the outer beam’s magnet is 15 mm (all lengths and spacings are measured from the magnet centers, as shown in Figure 1).

**Figure 24:**
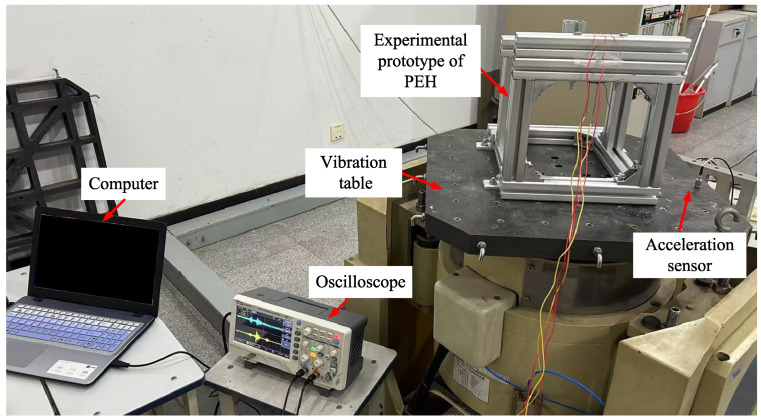
Experimental system for the PEH.

A test platform is established, as shown in Figure 24, where the experimental prototype of the PEH is fixed onto a vibration table. An excitation signal generated by a signal generator is amplified by a power amplifier to drive the vibration table into harmonic vertical motion. The base acceleration of the vibration table is measured by an acceleration sensor mounted on its surface. Under external excitation, the PEH produces mechanical vibrations, and its output voltages are monitored by an oscilloscope and finally input into a computer for data processing and analysis. The root mean square (RMS) values of the vibration table’s acceleration are set to 2 $\;m/s^{2}$ and 6 $\;m/s^{2}$ (corresponding to the amplitudes of 2.828$\;m/s^{2}$ and 8.485$\;m/s^{2}$, respectively). The experiment employs a frequency-sweep method, with a frequency range of 6–30 Hz and a sweep rate of 0.02 Hz/s.

**Figure 25:**
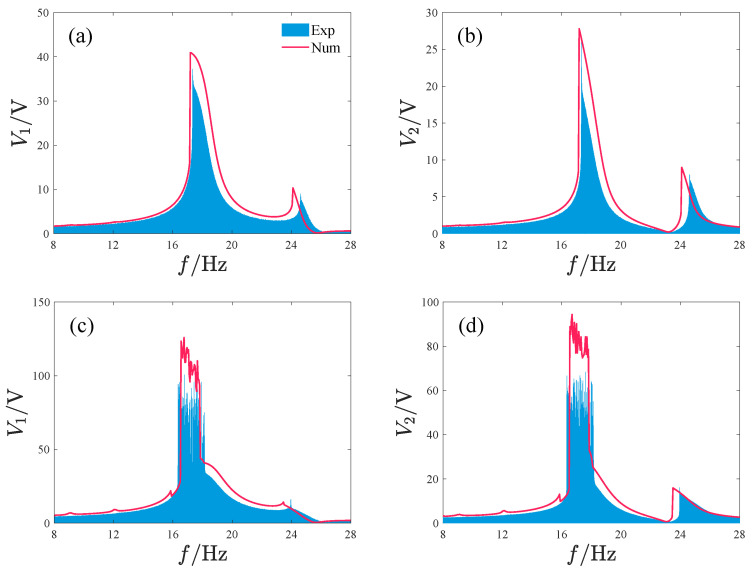
Comparison between the simulation and experimental results of the repulsive-type PEH, where Exp denotes the experimental results and Num denotes the simulation results: (**a**,**b**) $A=2.828\;m/s^{2}$. (**c**,**d**) $A=8.485\;m/s^{2}$.

**Figure 26:**
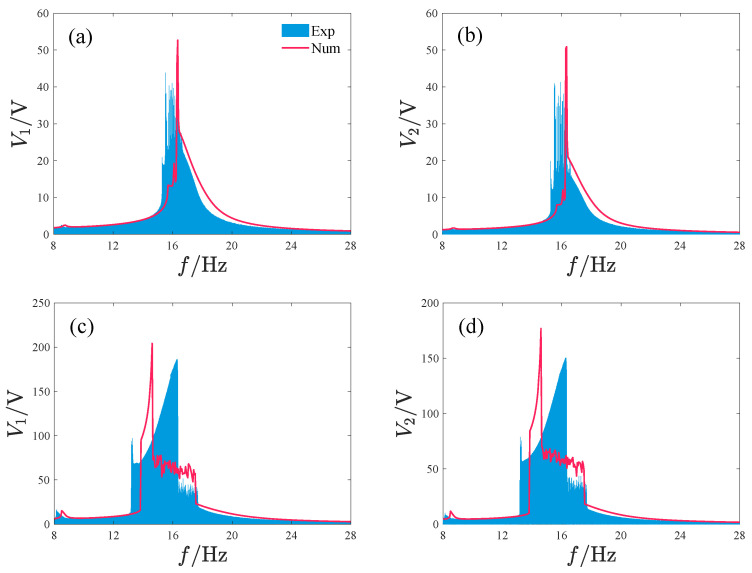
Comparison between simulation and experimental results of the attractive-type PEH, where Exp denotes the experimental results and Num denotes the simulation results: (**a**,**b**) $A=2.828\;m/s^{2}$. (**c**,**d**) $A=8.485\;m/s^{2}$.

Figure 25 and Figure 26 give the comparison between the simulation and experimental results of the repulsive-type and attractive-type PEHs, respectively. The simulation and experimental results of repulsive-type PEH show excellent agreement. For the attractive-type PEH, the two sets of data are highly consistent in power amplitude, variation trend, and peak frequency band, with only minor local discrepancies. These mainly result from two aspects: first, the magnetic dipole model used in the simulation for computational simplification does not fully consider the influence of actual magnet volume distribution on the magnetic field; second, the performance dispersion of the practical piezoelectric materials and the bonding state between the piezoelectric layer and substrate cannot fully match the ideal assumptions in simulation. In future research, we will further reduce such discrepancies by optimizing the magnetic model and accurately characterizing material parameters.

## 6. Conclusions

This paper investigates a dual-oscillator PEH incorporating an external magnet. Both repulsive and attractive interactions between the two oscillators are examined through simulation and experimental studies. The influence of key parameters on PEH’s static and dynamic characteristics, as well as on its energy harvesting performance, is analyzed. The main conclusions are as follows:

The repulsive-type PEH exhibits bistable characteristics. At small excitation acceleration amplitudes, the peak response is a periodic motion in which the two oscillators vibrate in phase. At higher amplitudes, the response is dominated by one oscillator vibrating with large amplitude and the other with small, and non-periodic motion may also occur. A larger external-magnet spacing facilitates improved bandwidth and average power output. However, beyond an optimal distance, performance gradually declines. The moving-magnet spacing has a negligible effect on the main frequency band. Increasing the external-magnet spacing reduces the peak frequency of the small-amplitude response, while the large-amplitude response frequency remains largely unchanged.

The attractive-type PEH is bistable at small external-magnet spacings and becomes monostable as the spacing increases. The moving-magnet spacing does not affect the static equilibrium. The peak response is primarily in-phase periodic vibration, with non-periodic motion possible at higher excitations. A combination of smaller moving-magnet spacing and larger external-magnet spacing is more favorable for achieving higher bandwidth and power output. Similarly to the repulsive-type case, performance within the optimal interval gradually decreases as the external-magnet spacing increases. The small-amplitude response frequency decreases with larger external spacing but increases with larger moving-magnet spacing; the large-amplitude response frequency is relatively insensitive to these changes.

The repulsive-type PEH possesses a wider potential well, resulting in superior performance under weak excitation and low vibration amplitudes. The attractive-type PEH, with its similar response form and potential well orientation, more readily forms large-amplitude vibrations and is better suited for environments with strong excitation.

Experimental results from the prototype show good agreement with simulations, particularly for output voltage and peak frequency.

## Figures and Tables

**Table 1:** Parameters of the PEH.

Parameters	Values
Width of substrate and piezoelectric layer *b*	5 mm
Length of the substrate of oscillator 1 $L_{S1}$	92 mm
Length of the substrate of oscillator 2 $L_{S2}$	108 mm
Thickness of the substrate $h_{S}$	0.3 mm
Density of the substrate $\rho_{S}$	$7800\;\mathrm{kg}/m^{3}$
Elastic modulus of the substrate $C_{S}$	170 GPa
Length of the piezoelectric layer $L_{P}$	20 mm
Thickness of the piezoelectric layer $h_{P}$	0.2 mm
Density of the piezoelectric layer $\rho_{P}$	7600 $\;\mathrm{kg}/m^{3}$
Elastic modulus of the piezoelectric layer $C_{11}$	56 GPa
The piezoelectric stress constant $e_{31}$	−9.41$\;C/m^{2}$
Relative permittivity $\varepsilon_{33}$	21 nF/m
Magnetic induction intensity *B*	1.25 T
Volume of the magnet *V*	$150\;{\mathrm{mm}}^{3}$
Mass of the magnet *m*	1.1 g
The moving-magnet spacing $d_{1}$	16 mm
The external-magnet spacing $d_{2}$	15 mm

**Table 2:** Comparison of the performance between the PEHs with and without external magnets.

PEHs	*A*/$m\cdot s^{-2}$	Fixed Magnet	BW/Hz	$P_{m}$/μW	Perf./μW·Hz	Improvement
The repulsive-type PEH	2.828	With	2	28.09	56.17	128%
		Without	1.6	15.41	24.66	
	8.485	With	6.65	34.71	230.85	75%
		Without	5	26.36	131.81	
The attractive-type PEH	2.828	With	4.5	19.73	88.76	364%
		Without	1.6	15.25	24.40	
	8.485	With	5.7	44.31	252.54	16%
		Without	4.95	44.15	218.54	

## Data Availability

The data that support the findings of this study are available from the corresponding author (Huabiao Zhang) upon reasonable request.

## Abbreviations

The following abbreviations are used in this manuscript:

PEH	Piezoelectric energy harvester
DOF	Degree of freedom
HBA	Harmonic balance analysis
